# Study and analysis of the optical absorption cross section and energy states broadenings in quantum dot lasers

**DOI:** 10.1016/j.heliyon.2022.e10587

**Published:** 2022-09-09

**Authors:** Mohammed S. Al-Ghamdi, Rafal Z. Bahnam, Ivan B. Karomi

**Affiliations:** a Department of Physics, Faculty of Science, King Abdulaziz University, P. O. Box 80203, Jeddah, 21589, Saudi Arabia; b University of Mosul, College of Education for Pure Science, Mosul, 41002, Iraq

**Keywords:** Absorption cross section, Linewidth broadening, Inhomogeneous broadening, Quantum dot laser

## Abstract

In this report, we measured experimentally the modal absorption spectra of the InP and InAsP quantum dot (QD) lasers using multi-section device technique. The optical absorption cross section (${\text{σ}}_{0}$) and inhomogeneous broadening for the ground state (GS) and excited state (ES) were analyzed and calculated theoretically from the absorption spectra. The results showed that the InP QD laser exhibited ${\text{σ}}_{0}$ to be $1.347\times 10^{-14}{\text{ ​cm}}^{2}.\text{eV}$ and $3.016\times 10^{-14}{\text{ ​cm}}^{2}\text{eV}$ for GS and ES respectively, whereas for the InAsP QD material it was found as $0.511\times 10^{-14}{\text{cm}}^{2}\text{eV}$ and $3.099\times 10^{-14}{\text{cm}}^{2}.\text{eV}$ for GS and ES respectively. Moreover, the inhomogeneous broadening in the GS increases from 35.6 eV to 63.6 eV when As was added to InP QD, similarly, the inhomogeneous broadening of ES increases from 46.9 eV to 103.8 eV. The alloying InP QDs with arsenic decreases the ${\text{σ}}_{0}$ of the ground state (lasing state) and increases both inhomogeneous and linewidth broadenings. This finding may help the grower to control the growth conditions and the molecule fractions of the crystal to improve the spectral properties of the optoelectronics devices.

## 1 Introduction

Quantum dot semiconductor structures have been of the most motivating materials in optoelectronic devices due to their unique properties such as spiked density of state, low temperature sensitivity [1], high data transmission rate [2], low threshold current [3] etc. These dots are typically grown by self-assembly method during the epitaxial process [4]. The crucial aspect of growing quantum dot materials is controlling the uniformity of the dots sizes. In fact, the grown dots in the stack system show a degree of irregularity in the dots sizes, this leads to the inhomogeneous broadening in the quantum dot system [5]. Therefore, it is significant to study the dot density distribution and broadening in these materials. Moreover, broadenings in quantum dot laser play a crucial role not only for the spectral characteristics of the laser [6], but also for its carriers dynamics [7]. Homogenous broadening, which arises from Heisenberg uncertainty principle, decreases the carrier scattering and relaxation oscillation frequency in QD lasers [8]. Additionally, the inhomogeneous broadening which is caused by fluctuations in QD sizes controls the threshold gain requirements in QD lasers [9]. Consequently, a number of studies have investigated the role of homogenous and inhomogeneous broadening in semiconductor quantum dot laser. However, most of these studies were theoretically conducted using set of rate equations for carriers and photons [10, 11, 12, 13]. On the other hand, calculating optical absorption cross section in semiconductor quantum dot laser is a key factor, which in turn provides a relationship between optical density of the sample and the concentration of quantum dots [14]. Many researchers have investigated absorption cross section in semiconductor materials such as colloidal InAs quantum dots [15] using model of small particle light absorption. Direct measurement of the optical absorption cross section of a single silicon quantum dot was reported in [16] using photon coating technique. The optical absorption cross section of QD laser was calculated in [17] by measured the modal optical absorption spectrum of a three-layer system of InAs quantum dots. The spectrally integrated cross section in InP/AlGaInP QD structures were determined in [18] from theoretical analysis of the ground state and exited state of the modal absorption spectra measured by segmented contact method. The aim of this article is to calculate the optical absorption cross section of the InP and InAsP QD lasers by simulating the modal absorption spectra of the samples which in turn will be experimentally measured by multi-section device technique. Additionally, inhomogeneous and linewidth broadenings were investigated in this study. The InP and InAsP QD lasers have showed a high optical quality [19] and high temperature laser operation [20] making them of significant interesting for further spectral investigations.

## 2 Sample structure and methodology

**Figure 1:**
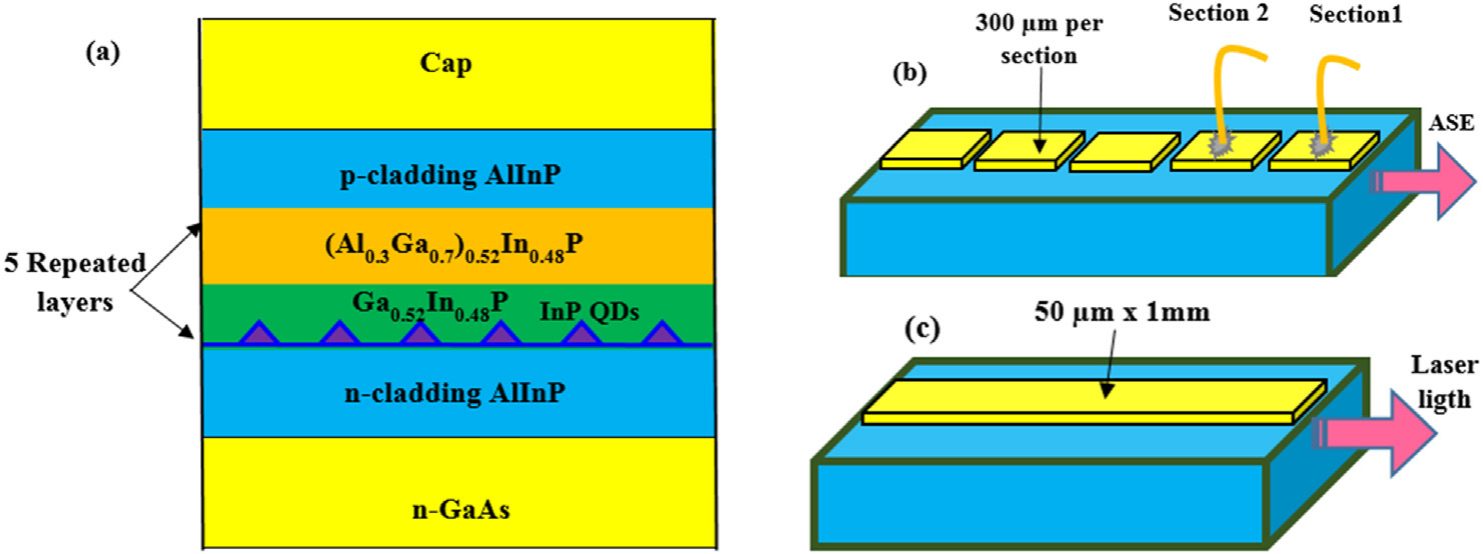
(a) samples structure, (b) multi-section device, (c) 1 mm laser device.

Two different QD laser samples were studied InP/GaAs and InAsP/GaAs QDs, both were grown under the same conditions and same structures. The samples were grown by organometallic vapour-phase epitaxy (OMVPE) on n-type on-axis GaAs (100) substrate, oriented 10^°^ off toward <111 > direction. The epitaxy was carried out at low-pressure flow OMVPE reactor using arsine AsH_3_ and phosphine PH_3_ as precursors of the group V elements. Identical structures but with either InAsP dot or InP dot active regions containing five layers of dot grown on (Al_0.3_Ga_0.7_)_0.51_In_0.49_P wells surrounding by 8-nm-thik of Ga_0.51_In_0.49_P quantum wells (QW) and separated by 16-nm-thik of (Al_0.3_Ga_0.7_)_0.51_In_0.49_P barriers. The waveguide cores were clad with 1μm of Al_0.51_In_0.49_P as shown in Figure 1a. For more details concerning growing processes and syntheses of the samples see [21]. The samples were processed into 50 μm wide and prepared as 300 μm multi-section devices for measuring the modal absorption using segmented contact method as shown in Figure 1b. In addition to 1 mm cavity length uncoated facets laser devices to measure the (power-current) curves and the emission spectra (see Figure 1c).

The absorption modal of the samples was measured using a multi-section device technique introduced in [22]. This technique involves measuring the amplified spontaneous emission (ASE) of section 1 of the device and the ASE of section 2 separately under specific pumped current. The modal absorption ($A_{m}$) can be calculated as [22]:

(1) $$A_{m}=\left(\frac{1}{L}\right)\ln\left(\frac{I_{ASE1}}{I_{ASE2}}\right)$$

where L is the section length of the multi-section device (in this case, L = 300μm), $I_{ASE1}$ and $I_{ASE2}$ represent the ASE spectra of section 1 and section 2 of the multi-section device, respectively. The modal absorption (*A*_*m*_) at a specified photon energy of optical mode propagation along the waveguide is known as [23]:

(2) $$A_{m}\left(E\right)=\frac{N_{i}}{W_{mod}}\sigma_{o}\left(E_{i}\right)$$

where $N_{i}$ is the number of dots per unit energy interval and $w_{mod}$ is the optical effective mode. The area under the modal absorption for a single transition gives the integrated optical cross section $\sigma_{0}\left(E\right),$ which is a distinct property of the dot and does not depend on homogeneous broadening. Accordingly, Eq. (2) can be rewritten as [23]:

(3) $$\oint A_{m}\left(E\right)\;d\left(E\right)=\frac{N_{dots}}{W_{mod}}\sigma_{o}\left(E\right)$$

**Figure 2:**
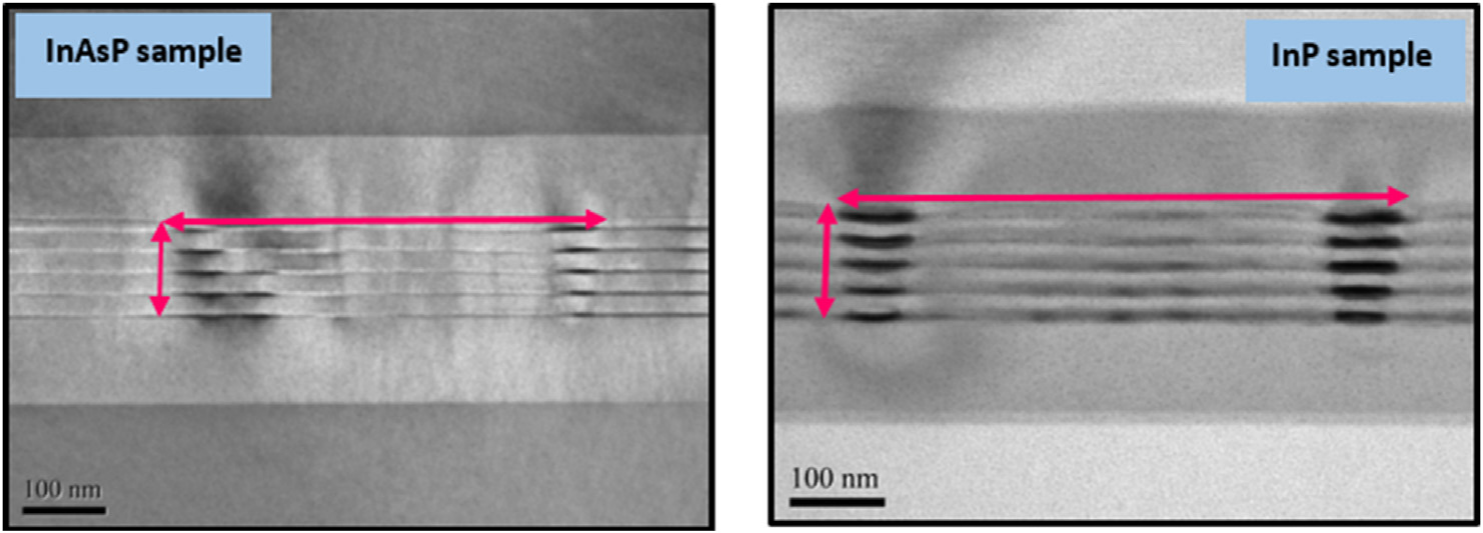
TEM images for the active region of InP and InAsP QD samples.

Figure 2 shows the cross section transmission electron microscopy (TEM) images that confirmed five layers of InP and InAsP QDs in wells. The dot density Ndots were estimated to be 1.15 × 10^10^ cm^−2^ for InP sample and 1.75 × 10^10^ cm^−2^ for InAsP sample.

## 3 Result and discussion

### 3.1 Laser results

**Figure 3:**
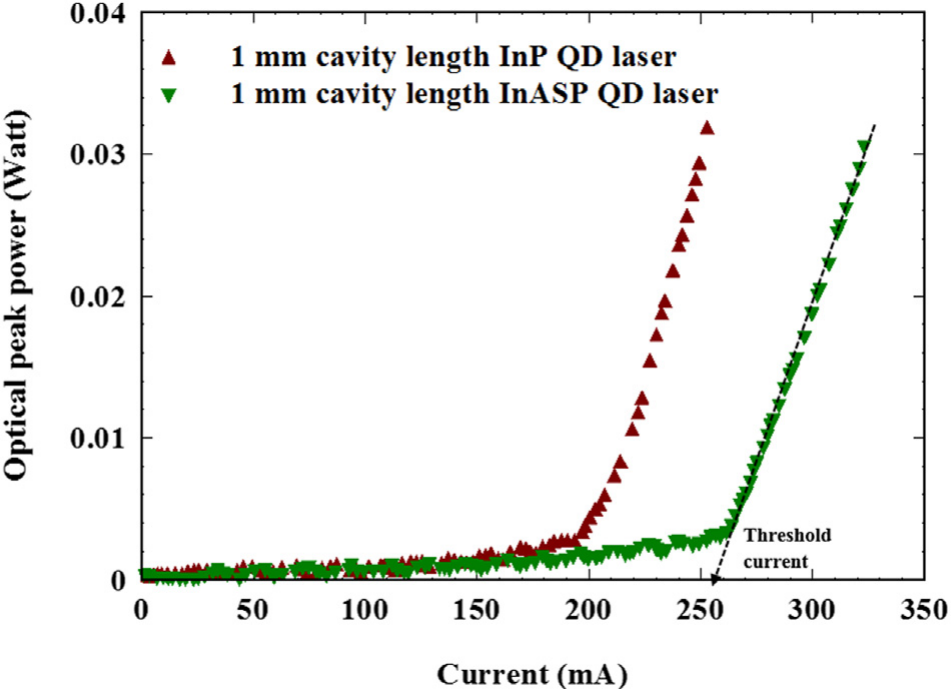
Optical power against current for the samples at room temperature under pulse mode operation.

The optical output peak power was measured by integrating sphere as function of the electrical current for both InP and InAsP 1 mm cavity length lasers and has been plotted in Figure 3. The samples were driven in pulse mode with duty cycle of 0.1 % to avoid the self-heating that causes redshift. The threshold current was calculated to be 195 mA and 270 mA for InP and InAsP lasers respectively (the threshold current determines by taking the interception of the straight line with x-axis). The larger threshold current of InAsP laser could be due to wider gain bandwidth for this material found in [7]. The slope efficiency of the samples (slope of the line above the threshold) found to be 0.63 W/A and 0.46 W/A for InP and InAsP receptively.

### 3.2 Amplified spontaneous emission and modal absorption

**Figure 4:**
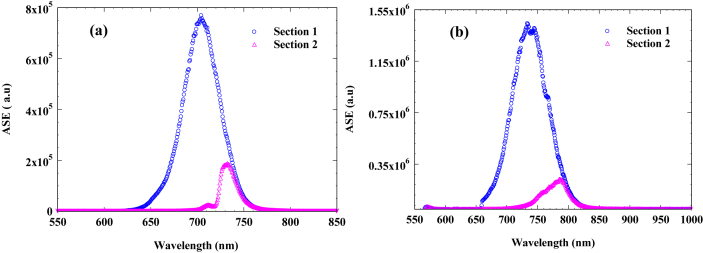
ASE for section 1 and section 2 measured by multi-section device technique for; (a) InP QD materials and (b) InAsP QD materials.

The amplified spontaneous emission (ASE) for section 1 and section 2 driven separately are depicted in Figure 4a and 4b for InP and InAsP QD materials respectively. It is possible to transform the ASE in Figure 4a and 4b to the modal absorption spectrum in real units by means of Eq. (1).

**Figure 5:**
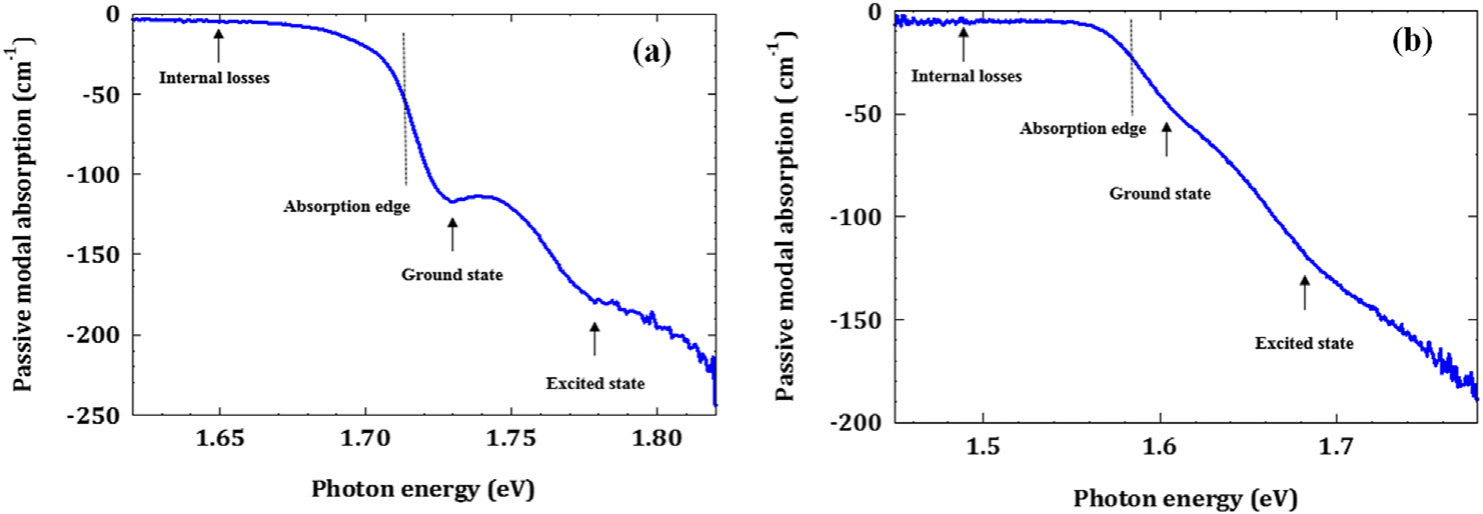
The modal absorption spectra for; (a) InP QD materials and (b) InAsP QD materials.

The modal absorption spectra of the InP and InAsP OD materials are plotted in Figure 5a and 5b, respectively. The important features of the modal absorptions are indicated in the Figures, namely, ground state (GS), excited state (ES), Absorption edges and internal optical loss region. It can be clearly seen that when the As was added to the InP, a red shift of around 60 nm is recorded in the absorption spectrum (shift of the absorption edge from 1.7158 eV to 1.5858 eV) and the ground state becomes less feature defined (the peak of state is not sharp as InP sample) and this leads for larger degree of inhomogeneous broadening in InAsP sample. The important parameters of the absorption spectra for both InP and InAsP QD lasers determined from Figure 5a and 5b are listed in Table 1.

**Table 1:** Important parameters of the absorption spectra for InP and InAsP QD sample.

Samples	Energy position (eV)		Amplitude (cm^−1^)		Absorption edge (eV)	Internal optical losses (cm^−1^)
	GS	ES	GS	ES		
InP	1.7290	1.7778	−116.5	−180.5	1.7158	3.8
InAsP	1.6096	1.6966	−50.1	−127.4	1.5858	5.2

### 3.3 Absorption spectra, optical cross section and inhomogeneous broadening

**Figure 6:**
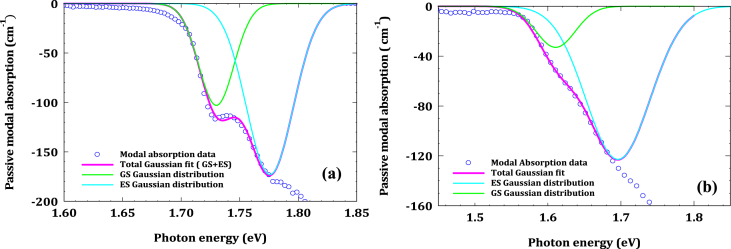
The modal absorption spectra with Gaussian distribution for GS and ES for; (a) InP QD materials and (b) InAsP QD materials.

The Gaussian distribution was applied for the GS and ES of both InAsP and InP samples, the fitting parameters used in the theoretical calculations are listed in Table 2 (we use EasyPlot 32 software for Gaussian fitting). Figure 6a and 6b represent the passive modal absorption spectra with the Gaussian fit of GS and ES for InP and InAsP QD materials respectively. The Gaussian distributions show the dot states distribution in the energy states and the area under the each fitted Gaussian represents the integration of Eq. (3). The $\left(w_{mod}\right)$ can be calculated from $w_{mod}=\frac{L_{z}}{\Gamma }$ [22], where L_z_ is the quantum well width (8 nm) and Γ is the optical confinement factor (0.02) for both samples. Hence, the optical absorption cross section was calculated from Eq. (3) in InP QD material as 1.347 × 10^−14^ cm^2^ eV and 3.016 × 10^−14^ cm^2^ eV for GS and ES respectively. Whereas, for InAsP QD materials, it is found 0.511 × 10^−14^ cm^2^ eV for the GS and 3.099 × 10^−14^ cm^2^ eV for the ES as it is listed in Table 3. The inhomogeneous broadening was calculated from the full width at half maximum (FWHM) of the Gaussian fit for GS and ES of the samples. The inhomogeneous broadening is calculated as 35.6 meV and 46.9 meV for GS and ES respectively in InP sample, while it is found as 63.6 meV and 103.8 meV for GS and ES respectively in InAsP sample. The results indicate that the optical cross section decreases in the GS when the As was added to InP, and this could be due to decreases of the absorption amplitude in GS of InAsP sample. However, the value of the optical cross section of the ES almost remained the same for both samples. Furthermore, the inhomogeneous broadening in InAsP sample is more obvious than in InP for both GS and ES because of the irregularity in dot size distribution in InAsP sample as it shown in Figure 2.

**Table 2:** Fitting parameters used in Gaussian function for InP and InAsP QD samples.

Samples	Peak energy (eV)		Absorption amplitude (cm^−1^)		Stander deviation (meV)	
	GS	ES	GS	ES	GS	ES
InP	1.7301	1.7758	−103	−173	15	20
InAsP	1.6106	1.696	−33	−123	27	44

**Table 3:** The results of Gaussian fit for InP and InAsP QD samples.

Samples	Dot density per unit area (*N*_*dot*_) (cm^−2^)	Area under the curve (eV cm^−1^)		Optical Absorption cross-section ($\sigma_{0}$) × 10^−14^ (cm^2^ eV)		Inhomogeneous broadening (meV)	
		GS	ES	GS	ES	GS	ES
InP	1.15 × 10^10^	3.8727	8.6720	1.347	3.016	35.6	46.9
InAsP	1.75 × 10^10^	2.2334	13.5613	0.511	3.099	63.6	103.8

### 3.4 Emission spectra and linewidth broadening

**Figure 7:**
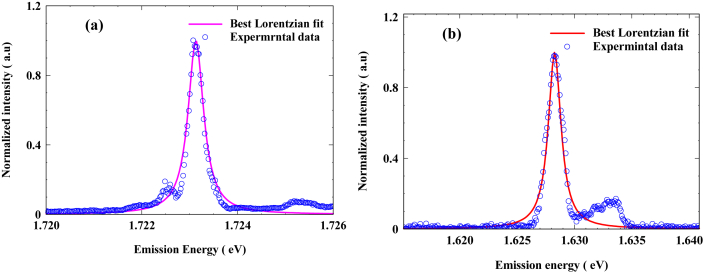
Emission spectra of 1 mm cavity length lasers with Lorentzian distribution for; (a) InP QD materials and (b) InAsP QD materials.

The lasing emission spectra of the samples were measured using optical spectrum analyzer. Figure 7a and b show the emission spectra of the GS energy of 1 mm cavity length laser of InP and InAsP respectively at room temperature taken slightly above the threshold current of each sample. We applied best Lorentzian distribution function on the experimental data in order to elicit the linewidth broadening which represents the full width at half maximum (FWHM) of the fitted function. The magnitude of the homogenous broadenings of the emission spectra for the ground state (lasing state) is found to reach 380 and 1480 μeV at room temperature for InP and InAsP respectively, which corresponds to electron lifetime of 866 fs ≥ and 222 fs ≥ calculated from Heisenberg uncertainty principle ((ΔEΔt≥ℏ2) where ΔE is uncertainty of the energy level and Δt is the life time of the carrier). Fitting parameters used in Lorentzian function and the results are listed in Table 4. To summarize the discussion above, the InAsP sample exhibited much longitudinal modes vibrating within the linewidth shape due to high value of homogeneous broadening in lasing emission by around 4 times in comparison to InP sample. additionally, the electron lifetime in InAsP sample is shorter than it in InP. This makes InAsP good candidate in passive integrated materials where fast recover time is required [24, 25]. High degree in the broadening of spectrum line in InAsP QD could be due to high coulomb carrier-carrier correlation in this material [10].

**Table 4:** Fitting parameters used in Lorentzian function and the results for InP and InAsP QD samples.

Samples	Lorentzian distribution parameter			linewidth broadening line width (μeV)	Carrier lifetime (Ps)
	Peak energy (eV) GS	Normalized emission amplitude GS	Stander deviation (μeV) GS		
InP	1.72313	1	198	380	0.866 ≥
InAsP	1.62828	1	670	1480	0.222 ≥

## 4 Conclusion

By experimentally measuring the modal absorption of the QD materials and using Gaussian distribution, we calculate the optical cross section and the inhomogeneous broadening in InP and InAsP QD lasers for both ground stat and excited state. Moreover, using Lorentzian distribution for laser ground state emission spectra, we have calculated the linewidth broadening and carrier life time for InP and InAsP QD laser. Adding As to InP QD material decreases the optical absorption cross section for the GS from $1.347\times 10^{-14}{\text{cm}}^{2}\text{eV}$ to $0.511\times 10^{-14}{\text{cm}}^{2}\text{eV}$ whereas, the ES showed no change (still approximately $3.099\times 10^{-14}{\text{cm}}^{2}\text{eV}$). Moreover, the inhomogeneous broadening and linewidth broadening of the ground state increase by 28 meV and 1.1 eV respectively, and the carrier lifetime reduces from 0.866 Ps to 0.222 Ps when As was doped to InP QD material. This study could pave the path to measured curtail limitation parameters of the OD materials used as an active region n of semiconductor lasers by only measured the absorption emission spectral of these materials. As a final point, the results of such studies could help the grower to control the growth conditions and the molecule fractions of the crystal to improve the spectral properties of the optoelectronics materials, hence, controlling the broadening in the laser materials for developing optimum optoelectronic devices for specific applications.

## Declarations

### Author contribution statement

Mohammed S. Al-Ghamdi; Performed the experiments. Rafal Z. Bahnam: Analyzed and interpreted the data; Contributed reagents, materials, analysis tools or data. Ivan B. Karomi: Conceived and designed the experiments; Wrote the paper.

### Funding statement

Mohammed S. Al-Ghamdi was supported by Deanship of Scientific Research (DSR) at King Abdulaziz University, Jeddah (Solid State Lighting) [(K/001/438)].

### Data availability statement

Data will be made available on request.

### Declaration of interest’s statement

The authors declare no conflict of interest.

### Additional information

No additional information is available for this paper.
